# Process Parameter Optimization for Enhanced Compressive Strength of FDM-Printed ABS and Glass-Fibre-Reinforced ABS Using RSM and Metaheuristic Algorithms

**DOI:** 10.3390/polym18192398

**Published:** 2026-10-01

**Authors:** Jaimon Dennis Quadros, Yakub Iqbal Mogul, Ganesan Subramanian, Ma Mohin, Suhas Poojary, Krishan Jones, Joseph Horne, Hanumantharaya Rangaswamy, Prashanth Thalambeti, Mohammad Shohag Alam

**Affiliations:** 1 Centre for Advanced Manufacturing, National Centre for Motorsport Engineering, School of Engineering and Built Environment, University of Greater Manchester, Bolton BL3 5AB, UK; j.quadros@greatermanchester.ac.uk (J.D.Q.); y.mogul@greatermanchester.ac.uk (Y.I.M.); k.jones2@greatermanchester.ac.uk (K.J.); j.horne@greatermanchester.ac.uk (J.H.); 2 Department of Computer Studies & Engineering, Symbiosis International University Dubai, Dubai Knowledge Park, Dubai P.O. Box 502930, United Arab Emirates; 3 Symbiosis International (Deemed University), Pune 412115, India; 4 Department of Mechanical Engineering, School of Engineering and Built Environment, University of Greater Manchester, Deane Road, Bolton BL3 5AB, UK; m.mohin@greatermanchester.ac.uk; 5 Department of Mechanical Engineering, Sahyadri College of Engineering & Management, Mangalore, Affiliated to Visvesvaraya Technological University, Belagavi 575007, India; 6 School of Mechanical Engineering, REVA University, Bangaluru 560064, India; hanumantharaya.r@reva.edu.in; 7 Department of Mechanical Engineering, Dr. H. N. National College of Engineering, Bengaluru 560070, India; trprashanth1977@gmail.com; 8 Faculty of Textile Technologies and Design, Istanbul Technical University, Gümüssuyu, Istanbul 34437, Turkey; alam@itu.edu.tr

**Keywords:** 3D printing, optimization, compression strength, ABS, ABS-GF, wall thickness, layer height, infill density

## Abstract

This study systematically investigates the effect of three Fused Deposition Modelling (FDM) printing parameters, i.e., wall thickness, layer height, and infill density, on the compressive behaviour of FDM-printed acrylonitrile butadiene styrene (ABS) and glass-fibre-reinforced ABS (ABS-GF). A Response Surface Methodology (RSM)-based Central Composite Design (CCD) comprising twenty experimental runs was employed to execute the experiments and develop regression models. The statistical significance of the process variables was determined through Analysis of Variance (ANOVA). Additionally, three nature-inspired optimization techniques, namely, the Firefly Algorithm (FA), Big Bang–Big Crunch (BB-BC), and Grey Wolf Optimization (GWO), were applied to identify the optimal printing conditions for achieving maximum compression strength. Experimental results demonstrated that glass-fibre-reinforced ABS exhibited superior compressive strength, greater load-carrying capacity, and more favourable deformation characteristics compared to neat ABS. Among the investigated parameters, infill density was found to be highly influential, as it controlled the internal porosity and structural continuity of the printed parts. The quadratic regression models developed exhibited excellent prediction accuracy, with coefficients of determination exceeding 95% for ABS and 97% for ABS-GF. Optimization results showed that the GWO algorithm achieved the highest fitness value of 97.15% and 98.48% for ABS and ABS-GF, respectively. The proposed optimization approach provides an effective framework for optimizing the compressive behaviour of FDM-printed polymer composites, thereby offering valuable guidance for the design and manufacture of high-performance engineering components through additive manufacturing techniques.

## 1. Introduction

Additive manufacturing (AM), especially fused deposition modelling (FDM), has become a widely used manufacturing technique due to its ability to produce complex geometries with its low equipment and material cost, ease of operation, and compatibility with a wide range of engineering thermoplastics and their composites [1]. Unlike traditional subtractive manufacturing techniques like CNC milling or turning, which produce components by removing material from solid billet, these techniques are generally constrained by factors including tool accessibility, fixture requirements, and machining waste. Contrastingly, FDM enables the fabrication of complex and customized geometries, including internal lattice and variable infill patterns, while simultaneously reducing material wastage, tooling costs, and overall lead time [1]. Among the thermoplastics used in FDM, acrylonitrile butadiene styrene (ABS) is extensively adopted because it can be processed with relative ease, low cost, and compatibility with composite fabrication [1,2]. However, the mechanical properties of pure ABS are limited to relatively low stiffness, process-induced anisotropic behaviour, and strong dependence on interlayer bonding. These disadvantages limit the applicability of ABS in high-performance engineering components. To address these limitations, fibre reinforcement strategies, especially those involving glass fibre (GF) and carbon fibre (CF), have been extensively investigated. Glass-fibre-reinforced ABS (ABS-GF) offers cost-effective improvement in stiffness and strength, whereas carbon fibre-reinforced ABS (ABS-CF) provides improved thermal stability and mechanical performance [3]. Hybrid composite systems integrated with GF and CF have attracted significant attention for their potential to exploit the synergetic benefits of both reinforcements. Nevertheless, recent findings emphasize that the mechanical performance of such systems is highly dependent on fibre matrix interaction, distribution, and processing conditions rather than fibre type alone [1,4]. These observations suggest that the effectiveness of any reinforcement is governed by complex process–structure–property relationships.

In FDM printing, there are multiple process parameters, such as wall thickness, raster angle, layer height, printing speed, extrusion temperature, infill density, etc., that play an important role in determining the mechanical behaviour of the final printed parts [2,3,4]. These parameters influence various factors like interlayer adhesion, porosity, fibre orientation, etc., all of which contribute to the anisotropic nature of fibre-reinforced printed parts [5,6]. Although fibre reinforcement has been widely adopted to improve the mechanical performance, weak interlayer bonding and fibre matrix interface limitations remain key challenges, often resulting in premature failure mechanisms such as delamination, fibre pull-out, and crack propagation. Despite significant research progress in FDM process optimization and material characterization, existing studies have focused on individual parameters or tensile/impact properties of ABS and its fibre-reinforced variants, with comparatively less attention given to the compressive behaviour of printed parts. Unlike tensile loading, compressive loading is governed by complex failure mechanisms, including interlayer debonding, densification, etc., which are highly sensitive to process parameters and reinforcement characteristics. The current study, therefore, aims to systematically investigate the compression behaviour of ABS and ABS-GF composites manufactured using the FDM technique. The following section reviews the existing literature with particular emphasis on process parameters, mechanical properties, commonly used printing materials (such as ABS, PETG, and PLA), fibre reinforcement strategies, and optimization approaches, thereby identifying the gaps that motivate the present study.

Fused deposition modelling (FDM) is a material extrusion additive manufacturing process in which thermoplastic material is softened, extruded through a heated nozzle and deposited layer by layer to form a component [2,5]. Several FDM parameters strongly influence the mechanical response, as they define both the geometry and quality of the printed structure. Chaudhry et al. [7] investigated the influence of different process parameters on the quasi-static behaviour of FDM-printed thermoplastic polyurethane. Their study confirmed that layer height had the strongest influence under quasi-static loading conditions, while infill density played a dominant role under high-strain loading conditions Using a similar parameter-driven response surface methodology (RSM) optimization approach, Kumaresan et al. [8] investigated the effects of infill pattern and raster angle on the mechanical properties of FDM-printed PETG. Their findings revealed that concentric infill patterns produced the highest tensile strength, while a cubic infill pattern at a 45° raster angle gave the best compressive performance. Eksi and Karakaya [9] analyzed the tensile behaviour of FDM-printed PLA using the Taguchi technique and found that infill density was the most influential parameter affecting tensile strength, while layer thickness had a greater effect on elongation at break. The regression models developed were able to predict tensile strength with reasonable accuracy. Beyond layer height and infill density, Rodriguez et al. [10] found that raster alignment with loading direction, combined with a negative air gap, improved both the stiffness and strength of ABS and PLA printed parts. Too et al. [11] demonstrated that increasing the air gap improved the porosity and consequently reduced the compression strength. Similarly, Dawoud et al. [12] reported that negative air gaps significantly improved the mechanical properties of ABS, although raster angle exerted greater influence under positive air gap. Hernandez et al. [13] examined the effect of build orientation on FDM-printed ABS and found that although its influence on tensile strength was minor, it had a significant impact on compressive and flexural properties. Overall, these findings confirm that infill density, layer height, and raster angle are consistently significant parameters across materials, while air gap and build orientation govern porosity and anisotropic failure behaviour rather than stiffness and strength.

Fibre-reinforced filaments have drawn significant attention, with carbon-fibre-reinforced PLA being investigated for tensile strength, wherein layer height and bed temperature were found to be more influential than extrusion temperature or print speed (Muhamedagic et al. [14]). Early foundational work conducted by Sood et al. [15] demonstrated that various FDM parameters, namely, layer thickness, raster angle, infill width, and air gaps, tend to influence microstructure and strength, linking partial fibre debonding to buckling failure. Radadiya and Gandhi [16] examined ABS alongside ABS-CF, ABS-GF and multi-fibre ABS and concluded that fibre type alone does not determine performance; rather, FDM parameters like raster angle, layer height, build orientation and fibre arrangement effectively influence how reinforcement contributes to load transfer. Aravind et al. [17] reported that ABS-GF exhibited the highest flexural and impact strengths among ABS, ABS-GF and ABS-GF-CF configuration. This suggests that adding fibres or combining reinforcement types can increase interlaminar complexity and the likelihood of delamination when stress transfer between fibres and the matrix is not efficient. Similarly, Dhandapani et al. [18] demonstrated that ABS-GF and ABS-GF-CF provide distinct advantages in terms of wear behaviour, indicating that the selection of reinforcement system depends on specific properties rather than fibre stiffness alone. Yet another study by Bas-Bolufer et al. [4] proved that glass fibre reinforcement improves ABS only when the fibre phase is properly aligned, bonded and integrated within the printed structure. Collectively, these findings indicate that both reinforcement strategy and process parameters play a critical role in determining the mechanical performance of FDM-printed composites.

Several microstructural factors govern the mechanical performance of FDM-printed parts, among which interlayer adhesion and porosity are widely recognized [19]. Studies on PLA and PLA-CF have shown that infill density and porosity distribution jointly influence the tensile and fracture response of the printed components [20]. Tekinalp et al. [21] demonstrated that the incorporation of carbon fibres into ABS improved stiffness and strength despite an increase in the porosity, indicating a close relationship between reinforcement and void content. Benal Dias Gonçalves et al. [22] further reported that inadequate bonding between the ABS matrix and fibre reinforcement reduces stress transfer, leading to failure mechanisms such as fibre pull-out, matrix cracking, local debonding and delamination. Additionally, process parameter optimization of FDM, including layer height, printing speed, and bed temperature, have been shown to minimize void formation and improve the interlayer fusion [23]. Liau et al. [24] observed that the crystallinity and appropriate bed temperature significantly improved the porosity, thereby affecting mechanical strength, of the FDM-printed parts. Furthermore, vibration-assisted FDM prints have been reported to further reduce porosity and improve ductility, at the expense of surface roughness [25]. Similarly, Kevlar fibre reinforcements embedded into the ABS/PLA composites were found to enhance the interfacial bonding and thermo-mechanical stability [26,27]. Overall, these findings confirm that the failure of FDM-printed parts is predominantly governed by the complex interaction of porosity, interlayer adhesion, and process-induced anisotropy. Table 1 summarizes the studies that have implemented various neural networks and response surface methodology (RSM) techniques on the mechanical properties of FDM-fabricated parts.

While considerable research is carried out on ABS and glass-fibre-reinforced ABS, most studies have primarily focused on tensile, impact, hardness, and flexural properties, with compression behaviour receiving comparatively limited attention. This represents an important research gap, as many practical FDM applications, including load-bearing brackets, enclosures, jigs, and fixtures, are subjected to compression loading. The failure of such components is primarily governed by interlayer debonding, densification, and the buckling of interlayer architecture, which cannot be predicted through tensile testing. This makes compression strength a critical design parameter that cannot be directly inferred from tensile properties due to the inherent anisotropic nature and interlayer architecture of the printed parts. Furthermore, while the RSM technique has been widely adopted across different FDM optimization studies, the use of a Central Composite Design (CCD) framework to model and optimize compression strength as a function of wall thickness, layer height, and infill density has not been adequately explored, specifically for a comparative study involving both ABS and ABS-GF materials. This gap forms the basis of the present study, which establishes a systematic process–property optimization framework using 20 RSM-based CCD experimental runs to enhance the performance of both materials under compressive loading. In addition, this study incorporates three optimization algorithms, namely, FA, BB-BC, and GWO, which, to the best of authors’ knowledge, have not been applied for optimizing the compression strength of FDM-printed ABS and ABS-GF. The integration of these algorithms with the RSM-based CCD modelling represents a novel methodological contribution, enabling a more robust and comprehensive optimization framework that extends beyond the conventional ANOVA-based approaches commonly reported in the FDM literature.

## 2. Materials and Method

### 2.1. Materials

**Figure 1:**
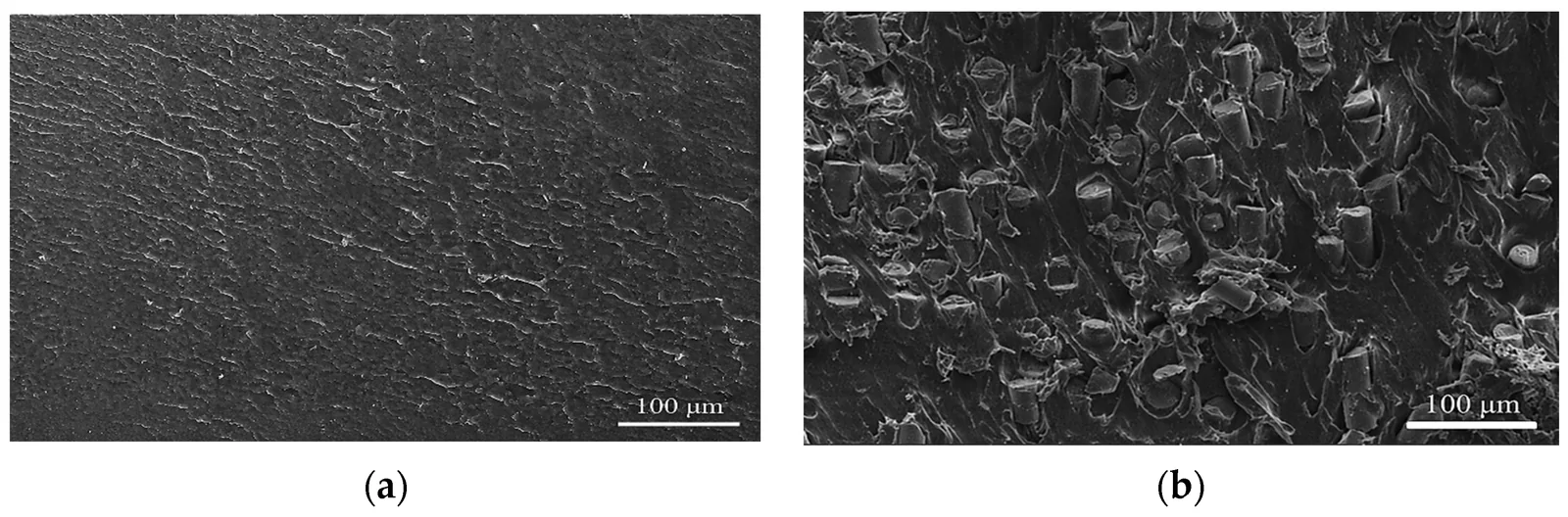
SEM images of (**a**) neat ABS and (**b**) ABS reinforced with short glass fibres [22,37].

The current investigation involved printing specimens for compression using Acrylonitrile Butadiene Styrene (ABS) and short glass-fibre-reinforced ABS (ABS-GF) filaments of 1.75 mm in diameter through the Fused Deposition Modelling (FDM) technique. Both types of filaments were commercially procured and used as received, without further processing. The ABS-GF filament consisted of E-glass fibres of approximately 13 μm initial fibre diameter and 3 mm fibre length, dispersed within the ABS matrix at a fibre volume of 7.5% (equivalent to ~15% by weight). Both types of filaments were stored under humidity-controlled conditions prior to printing to minimize moisture absorption. ABS-GF was produced through melt compounding, wherein E-glass fibres were dispersed with molten ABS in a twin extruder to achieve uniformity. The compounded pellets were then extruded through a single screw extruder (US Extruders, Inc., Westerly, RI, USA) to produce 1.75 mm diameter filament, followed by cooling and spalling. The microstructures of neat ABS and ABS-GF are provided in Figure 1. The individual mechanical and physical properties of glass fibres and ABS are presented in Table 2 and Table 3, respectively. The reported values in Table 2 and Table 3 correspond to standard ambient/room temperature conditions (23 ± 2 °C), consistent with polymer testing standards.

### 2.2. Fabrication and Experimentation

**Figure 2:**
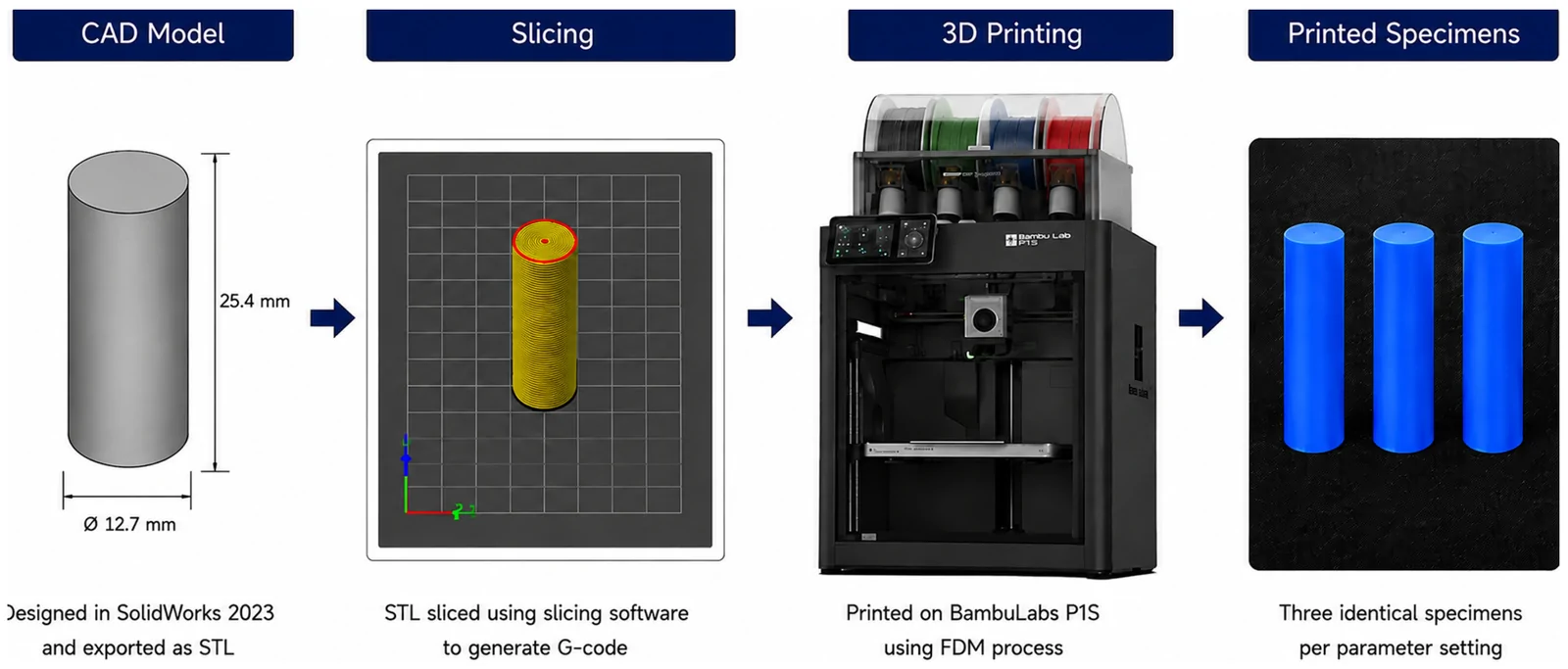
Workflow of FDM-printed specimens: from CAD models to finished parts.

**Figure 3:**
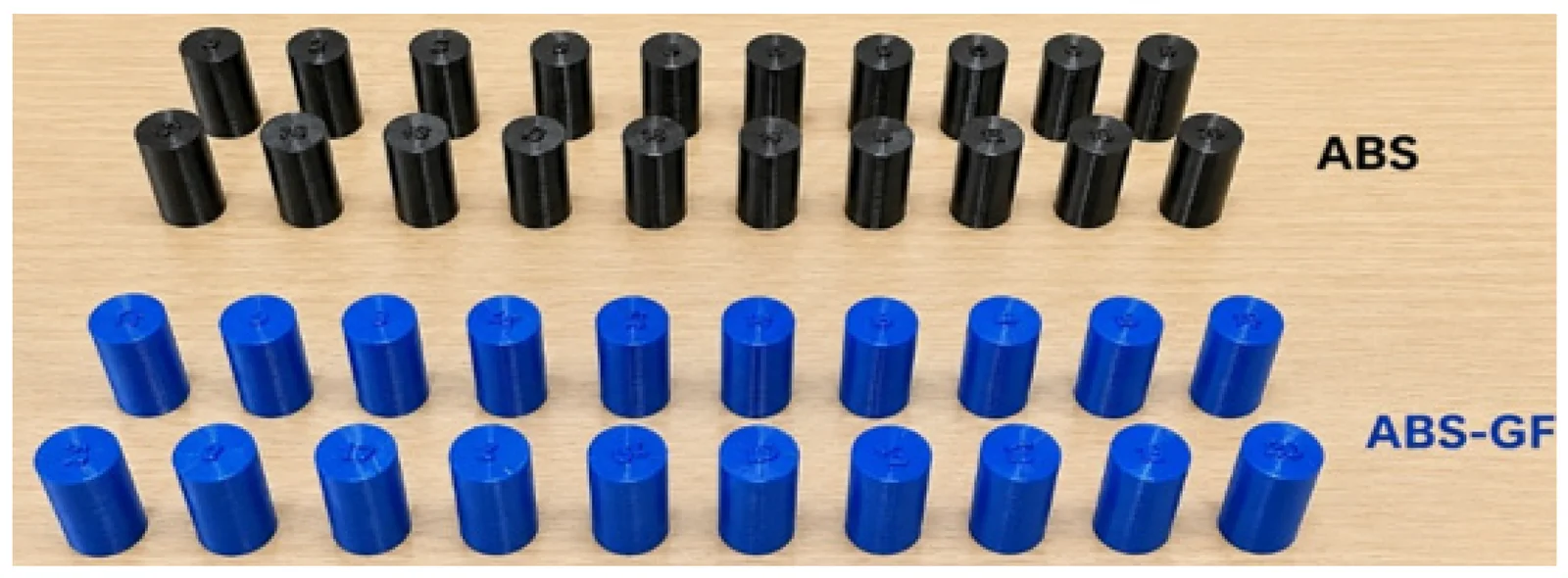
FDM-printed ABS and ABS-GF specimens for compression testing.

The geometries of the specimen complied with the ASTM D695 standards [36] for compression testing of rigid plastics. The CAD model of the test specimens as per ASTM standards was designed using the SolidWorks 2023 software V3 and subsequently converted to STL format. A Bambu Slicer 3D printing software version 2.1.0, which generated a G-code, was used to slice the STL file based on a specific set of printing conditions. The 3D printer used for fabricating the specimens was Bambu Labs PS1. The three main FDM process parameters examined in this study were wall thickness, layer height, and infill density. The parameters and their corresponding levels were selected based on previous research [28,29,30,31,32,33,34,35,36]. The wall-thickness selection was centred around two-times the nozzle diameter, i.e., 0.6 mm, ensuring shell integrity, while the layer height was set according to the standard resolution settings for the Bambu Lab PS1 printer used in this study. The infill density was selected based on values investigated in the literature for balancing the mechanical performance against material usage and print time. The interference of mechanical anisotropy was excluded by building the sample at a 0-degree raster angle with flat build orientation. A nozzle diameter of 0.6 mm, nozzle temperature of 250 °C, platform temperature of 100 °C, and print speed of 30 mm/sec was kept constant throughout the printing process. For each combination of process parameter, three identical specimens were fabricated and tested. The workflow process adopted for the fabrication of FDM-printed specimens is illustrated in Figure 2. The specimens were conditioned at room temperature under laboratory conditions for 24 h prior to testing to attain equilibrium moisture content. The reported strength of each experiment was calculated as the average of the measured values. The FDM-printed ABS and ABS-GF specimens for compression testing are shown in Figure 3.

**Figure 4:**
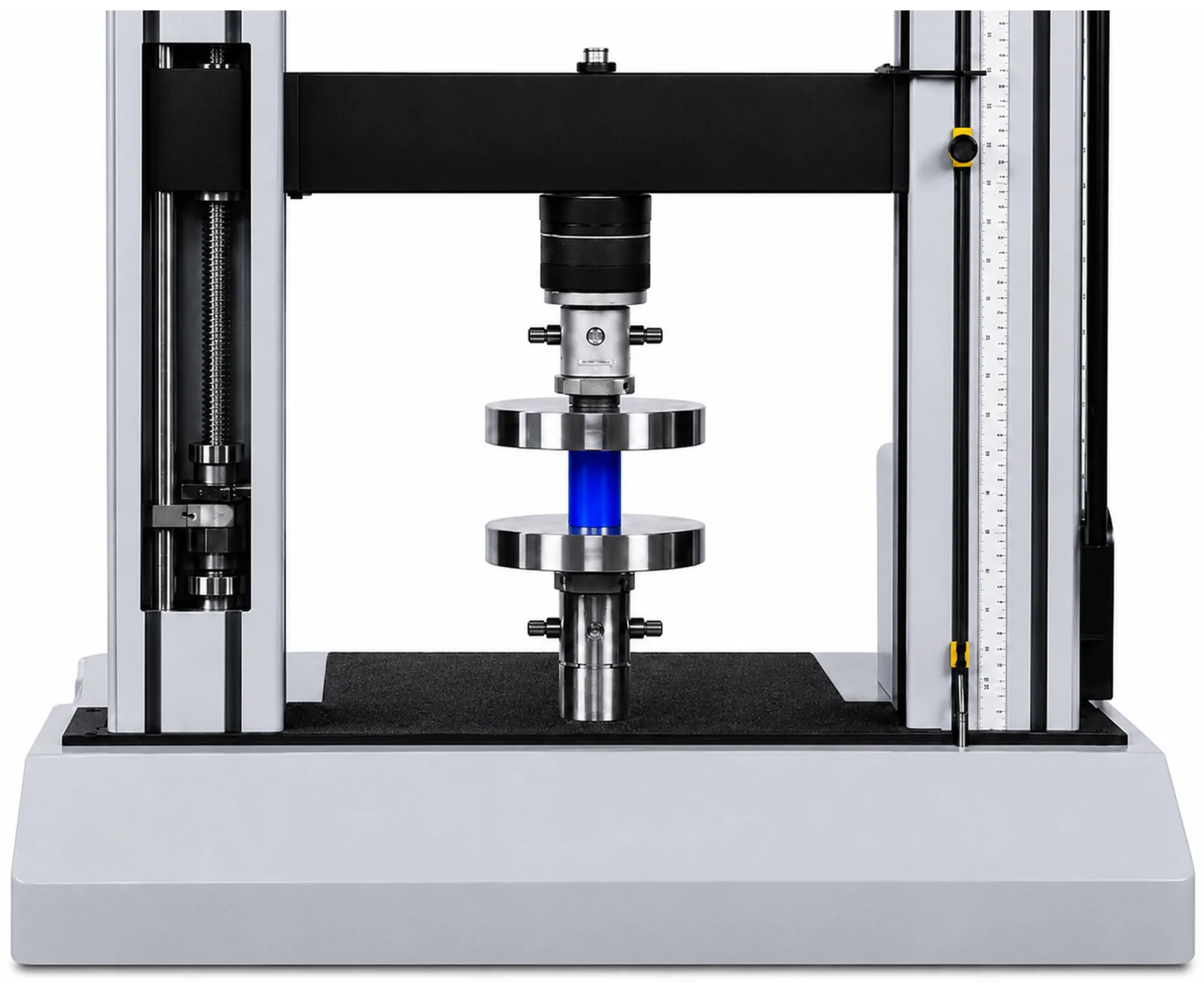
Compression testing of FDM-printed specimen.

The compression test was performed using an Instron Universal Testing Machine (UTM) equipped with a 10 kN load cell, following the ASTM D695 standard. The dimensions for the ASTM standard specimen are shown in Figure 2, while Figure 4 shows the setup for compression testing. The crosshead speed throughout the test was kept constant at 0.1 mm/min. The machine used in this study is calibrated to Class A as per ASTM E4, which specifies a load measurement accuracy of ±1% of the indicated force reading. The UCS represents the maximum load per unit area a material can withstand prior to failure. The UCS is determined by analyzing the peak point of the stress–strain curve during compression testing. Moreover, the stress–strain curve provided insights into other critical properties, such as compressive modulus, signifying the resistance of the material to elastic deformation when subjected to compressive force. The failure criteria were determined either by the appearance of crack propagation on the specimen surface or by cessation of the crosshead movement, i.e., the machine could no longer maintain the intended strain rate.

### 2.3. Experimental Design and Statistical Analysis

The Taguchi and response surface methodology (RSM) techniques are typical statistical modelling techniques for determining the influence of independent process variables on response variables [38,39,40]. For our study, the RSM-based Central Composite Design (CCD) was used to explore the impact of independent variables on the compression strength of ABS and ABS-GF. Three independent printing parameters/process variables were considered for experimentation. The process variables and their levels have been provided in Table 4. The CCD involved $2^{n}$ factorial runs, (2×n) axial runs, and $n_{c}$ centre runs [41]. The centre points generally determine the experimental error and evaluate the reproducibility of the data. On the other hand, the axial points maintain rotatability, ensuring that the variance of the model’s predictions remains uniform at all locations that are equally distant from the design centre [41,42]. The total number of experimental runs is calculated by using Equation (1), as shown below.

(1) $$N=2^{n}+\left(2\times n\right)+n_{c}=2^{3}+\left(2\times 3\right)+6=20$$

where n is the number of independent process variables. This indicated that the total experimental runs comprised 8 factorial runs, 6 axial runs, and 6 centre runs, which were used for the modelling and optimization process, as shown in Table 5. The obtained experimental data was run through the statistical Minitab 23 software. The primary objective of the study was to evaluate the influence of input variables on the response variable, i.e., ultimate compression strength (UCS), and to develop an empirical mathematical model using experimental data through batch testing. The model relates the response to three variables with a second-order polynomial expression provided in Equation (2).

(2) $$Y=b_{0}+\sum_{i=1}^{n}b_{i}X_{i}+\sum_{i=1}^{n}b_{ii}X_{i}^{2}+\sum_{i=1}^{n-1}\sum_{i+1}^{n}b_{ij}X_{i}X_{j}$$

where Y represents the response variable, $b_{0}$ represents the constant coefficient, $b_{i}$ represents the linear coefficient, $b_{ii}$ represents the interaction coefficient, $b_{ij}$ represents the quadratic coefficient, and $X_{i}$ and $X_{j}$ represent the coded value of factors.

### 2.4. Artificial Intelligence Tools for Optimization

**Figure 5:**
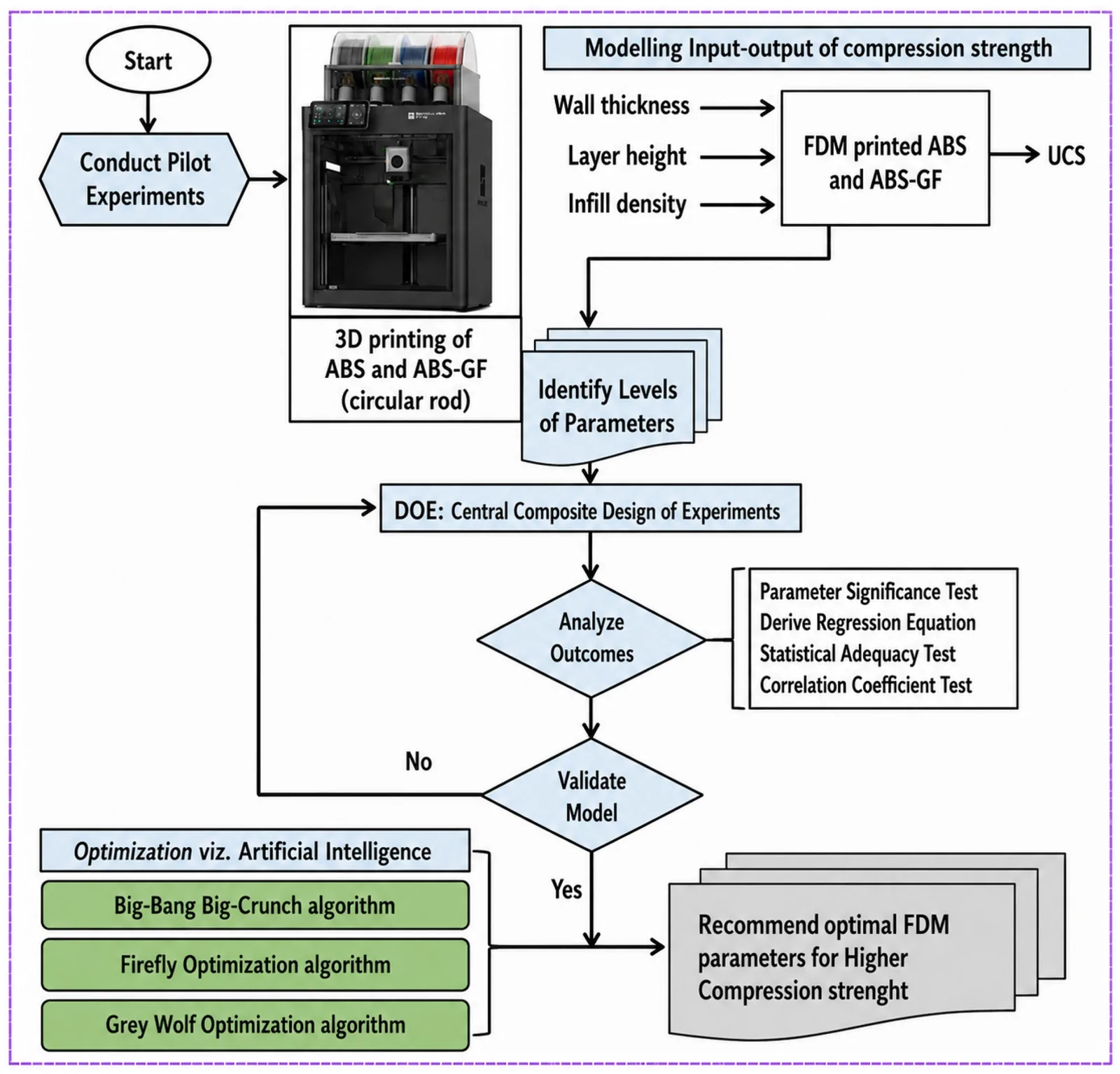
Framework for modelling and optimization of FDM-printed ABS and ABS-GF.

The experimental results obtained from CCD were subjected to statistical analysis. A regression equation/model was developed using the Minitab 23 statistical software. The adequacy of the model was evaluated through the coefficient of determination (*R*^2^), and the final model included all the terms, i.e., linear, quadratic, and interaction terms. The resulting empirical relationships were subsequently employed to identify the optimum printing parameter conditions for maximizing ultimate compression strength. The current work attempts to optimize the printing parameters using three heuristic search algorithms: the BB-BC (Big Bang–Big Crunch algorithm), FA (Firefly Algorithm), and GAO (Grey Wolf Optimization algorithm). It is important to note that the algorithm performance in terms of solution accuracy and computational time is highly dependent on the problem domain. Figure 5 shows the complete framework for modelling and optimization of the FDM-printed ABS and ABS-GF.

#### 2.4.1. Firefly Algorithm (FA)

Proposed by Yang in 2010, the FA, belonging to the swarm intelligence family, is inspired by the flashing behaviour and communication patterns of fireflies [43]. The FA is a technique that was utilized to optimize different problems associated with varying applications, such as engineering, image processing, economics, robotics, etc. The Firefly Algorithm employs a set of rules and assumptions derived from the flashing patterns and bioluminescent behaviour of fireflies to solve optimization problems [44,45].

- Fireflies are unisex; regardless of sex, they attract each other.
- Fireflies with lower intensity are attracted towards fireflies that emit stronger light, irrespective of their gender, thereby enabling an effective search for the most suitable partner. The fireflies exhibiting the highest brightness represent the optimal solution, while the degree of attraction between two fireflies diminishes as the separation distance between them increases, as described by Equation (3).

(3) $$I≺\frac{1}{r^{2}}$$

- The fireflies exhibit random movement when their brightness remains identical or equal.
- The absorption coefficient (γ) of the light travelling through a material with a light intensity of (r) from the source is given by Equation (4).

(4) $$I=I_{0}e^{-\gamma r^{2}}$$

- The brightest firefly travelling through its surroundings conducts a local search randomly. Resultingly, if firefly j is brighter than firefly I, firefly I will travel towards firefly j using Equation (5).

(5) $$x_{i}=x_{i}+\underset{=\beta }{\underbrace{\beta_{0}e^{-\gamma r^{2}}}}\left(x_{j}-x_{i}\right)+\alpha (\varepsilon ()-0.5)$$

where $\beta_{0}$ represents the attractiveness of $x_{j}$ at r=0, $I_{0}$ is the source light intensity, γ is the updating process when the distance between two fireflies is modified, α is the algorithm variable for random movement step length, and ε() is the random distribution vector ranging between 0 and 1.

#### 2.4.2. Big Bang–Big Crunch Algorithm (BB-BC)

The BB-BC is a unique algorithm that is gradient-free and based on a cosmological theory that suggests that the universe has two phases—the big bang (BB) and big crunch (BC)—proposed by Erol and Eskin [46]. When compared to the evolutionary genetic algorithm, the BB-BC achieves faster convergence requiring low computational effort and processing time [46,47]. The BB phase is characterized through disorder production and generation of randomness by energy consumption, resulting in particles being randomly distributed throughout the space. During the subsequent BC phase, these particles are replaced by the ones featuring better characteristics, thereby guiding the search towards an optimal solution [48]. The following steps are implemented to determine the solution for optimization problems using the BB-BC algorithm [46,48].

- The preliminary set of candidate solutions are randomly distributed according to the search space.
- The corresponding fitness value functions of the individual candidate solutions are then determined.
- Following this, a convergence operation is performed in which numerous input values are combined to obtain one output value. The global stiffness is then determined according to the centre of mass.
- As the optimization progresses, updated candidate solutions are generated by introducing random perturbations around the centre of mass, and their fitness values are subsequently evaluated.
- The algorithm determines whether the pre-defined termination condition was satisfied. If reached, the optimization process is terminated; otherwise, iterations continue until convergence is reached.

#### 2.4.3. Grey Wolf Optimization Algorithm (GWO)

Proposed by Mirijalilli et al. [49], the Grey Wolf Optimizer (GWO) models collaborative hunting through a hierarchy of grey wolf packs, which are designated as α, β, δ, and ω. The social organization of grey wolves is based on a pack system, where members are categorized into four levels based on their dominance and responsibilities [50]. Alpha (α) occupies the topmost position, potentially referred to as a leader, with a primary function of making decisions for the pack regarding hunting, sleeping, and wake-up time [51]. Note that (α) are the strongest and their decisions must be obeyed by other individuals according to social hierarchy. Acting as leaders, the β wolves support the α wolves in decision-making processes through continuous feedback and are also regarded as the most likely successors of the α wolves. The δ wolves follow the orders given by the α and β wolves and are often responsible for searching boundaries, encountering dangerous situations, and ensuring security.

In the GWO, a pre-defined population of grey wolves collaboratively explore the optimal solution in a multi-dimensional search space to locate the target solution [52]. Throughout the optimization process, the fitness of each grey wolf is computed based on its current position relative to its target (prey) within a search space. However, as the iteration progresses, the individual grey wolf tries to obtain the best solution by adjusting its position to a better position. It must be noted that the primary objective of the GWO is to identify the most efficient pathway for grey wolves to reach their prey. The process of searching for the optimal solution is carried out through the following four stages [49,50,51].

- ***Exploration:*** The wolves continuously regulate their position with respect to one another to track, chase, and surround the victim or prey.
- ***Encircling:*** The positions of grey wolves are updated based on three wolves (*α*, *β*, and *δ*) in a search space.
- ***Hunting:*** Through effective tracking skills, the grey wolves are capable of detecting the prey’s location. The *α* wolves lead the hunt, while *β* and *δ* tend to join them later. The positions for *ω* are adjusted with respect to the top three fittest individuals among *α*, *β*, and *δ*.
- ***Attacking the prey:*** The hunting process concludes only after capturing the prey and ceasing its movement.

## 3. Results and Discussion

In this section, the results of the experimental input–output data, the contribution of linear, square, and interaction variables, regression equations and their statistical adequacy, and the curvature effect analysis are discussed. The optimal combination of 3D printing parameters maximizing the compression strength of ABS and ABS-GF is determined and compared with the efficiencies of three computational modelling tools (FA, BB-BC, and GWO). Finally, the stress–strain curves analyzing the compression strength of both materials are discussed.

### 3.1. Compression Strength Behaviour for ABS and ABS-GF

**Figure 6:**
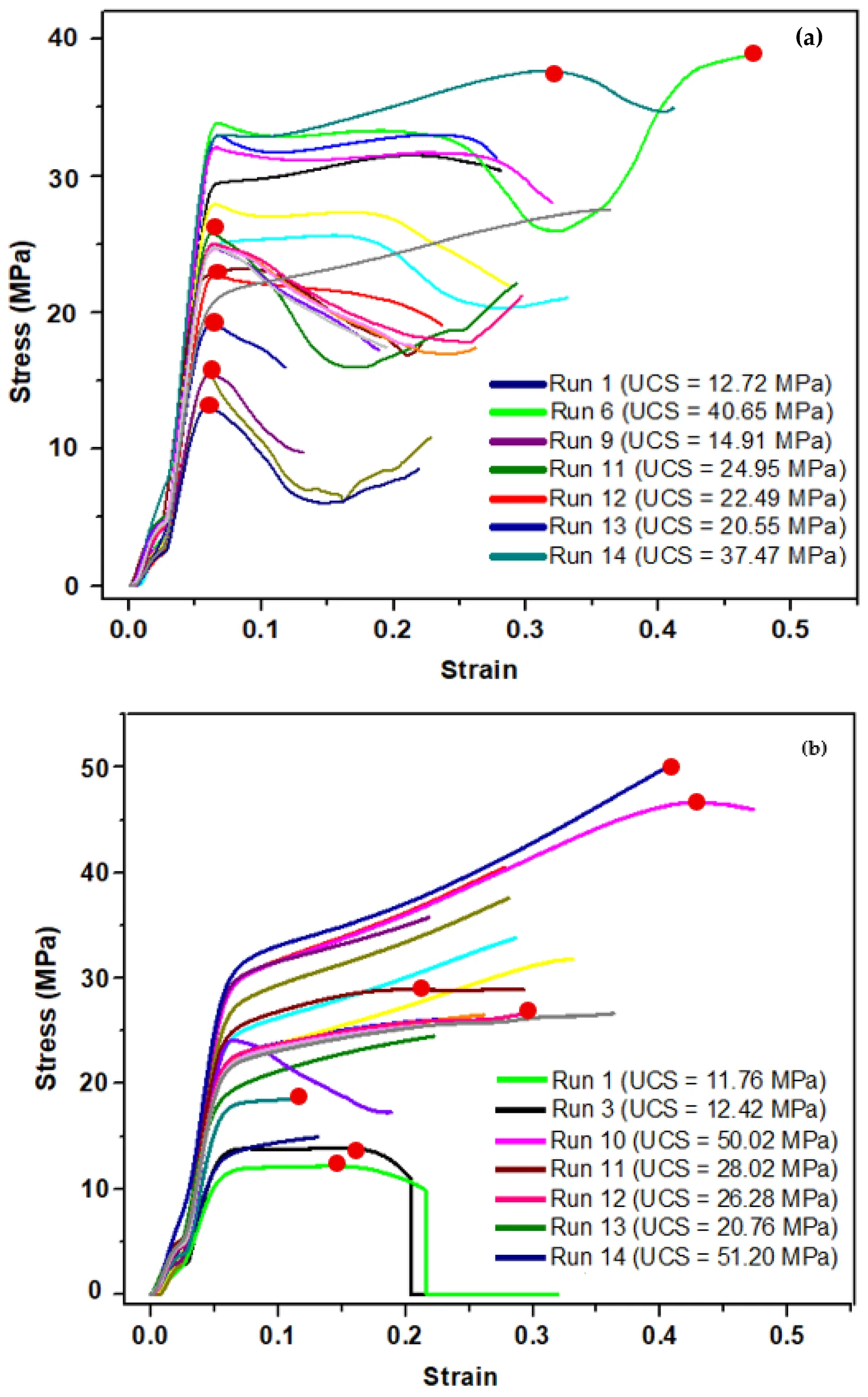
Stress–strain curves of FDM-printed (**a**) ABS and (**b**) ABS-GF samples under compressive loading, fabricated according to the CCD experimental runs.

The 20 curves shown in Figure 6 represent the stress–strain curves for FDM-printed ABS and ABS-GF samples, prepared according to the CCD experiments presented in Table 5. Figure 6a shows the stress–strain curves for compression behaviour for FDM-printed ABS samples. Red markers indicate the Ultimate Compressive Strength (UCS) points corresponding to different runs and were selected to represent the full spectrum of compression behaviour. This included the two highest, two lowest, and three intermediate UCS values. The remaining exhibited UCS values around the intermediate range and followed similar trends of the selected curves; therefore, they were omitted for clarity and discussion redundancy. All 20 samples exhibited similar characteristic behaviour, with an initial steep elastic rise up to a strain of 0.04–0.06, followed by peak stress representing the ultimate point. Beyond this point, the curves either plateaued, decreased, or, in some cases, showed a slight increase before failure. The highest UCS recorded was 40.65 MPa and 37.47 MPa for Runs 6 and 14, respectively, confirming that thicker walls, finer layers, and high infill density produced a compact and strongly bonded internal structure with minimum porosity. Both specimen curves show continuous strain hardening after the initial yield region, maintaining structural integrity. In contrast, the lowest strength was 12.72 MPa (Run 1) and 14.91 (Run 9), where low wall thickness failed to provide sufficient load-bearing capacity, leading to subsequent failure at low UCS. This behaviour is indicative of premature structural instability, resulting in reduced ability to withstand compressive loading [14]. This trend is complemented by axial points, where Run 13 with a minimum infill of *I_d_* = 4.77% yielded 20.55 MPa, while increasing it to a maximum of *I_d_* = 55.23% (Run 14) yielded 37.47 MPa, holding *W_t_* and *L_h_* constant at centre values, delineating infill density’s strong correlation with UCS. Run 11 and Run 12 achieved intermediate UCS values of 24.95 MPa and 22.49 MPa, respectively, showing early followed by gradual softening and plateau-like behaviour, highlighting progressive internal deformation rather than catastrophic failure. Both runs have the same values of *W_t_* and *I_d_* with maximum change in layer height, i.e., 0.0659 and 0.2341 mm, respectively, and very adverse effects on UCS, indicating that although the overall interlayer bonding was modified, the load-carrying capacity was moderate.

Figure 6b shows the stress–strain curves for compression behaviour for FDM-printed ABS-GF samples. Compared to plain/neat ABS, the GF-reinforced ABS samples show superior compression strength, with several curves showing continuous increases up to 30–51 MPa without an early plateau, indicating efficient load transfer between matrix and glass fibres. This behaviour also suggests strain-hardening behaviour before failure. Certain samples, notably Runs 1 and 3, displayed very low UCS values of 11.76 and 12.42 MPa, respectively, showing pronounced yielding followed by a sudden vertical drop to zero stress. This behaviour is attributed to the brittle nature and premature failure of the specimen, rather than gradual softening, and is likely due to localized buckling, layer delamination, and collapse of internal structure [53,54]. The highest compression strengths were observed for the sample Runs 10 and 14, i.e., 50.02 MPa and 51.02 MPa, respectively, which possessed higher infill density and thicker walls. The stress–strain curves are characterized by strain hardening, indicating excellent deformation capability and energy absorption characteristics. Runs 11, 12, and 13 represent intermediate UCS values 28.02 MPa, 26.28 MPa, 2076 MPa, respectively. The stress–strain curves here exhibit an initial elastic region beyond which stress values rise and approach a plateau prior to peak stress. This indicates progressive deformation and is typical of structures capable of redistributing compressive loads through localized deformation mechanisms [17,53]. Overall, the reinforcement of ABS with GF enhanced peak compression strength, and several stress–strain curves observed a more continuous rise in stress values as compared to the ABS curves. This behaviour indicates better load transfer and greater ability for the reinforced material to sustain deformation prior to failure.

### 3.2. Main Effect Parameter Analysis

**Figure 7:**
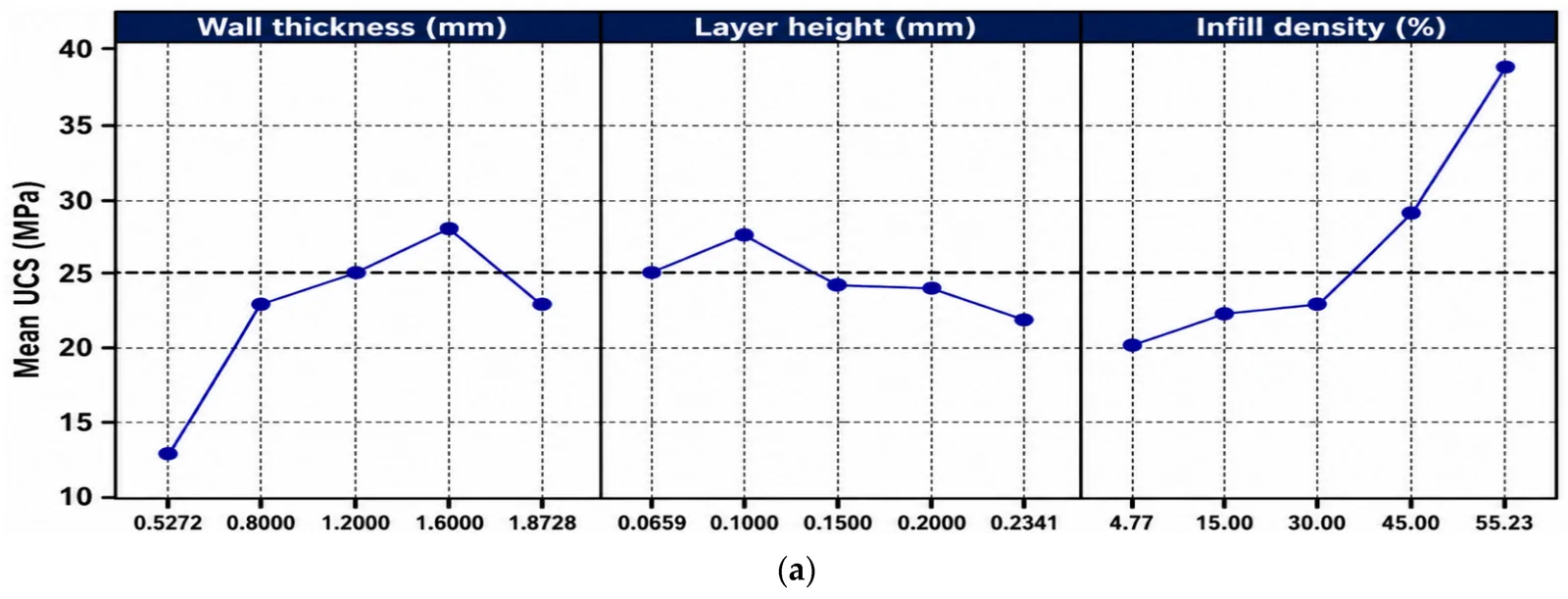

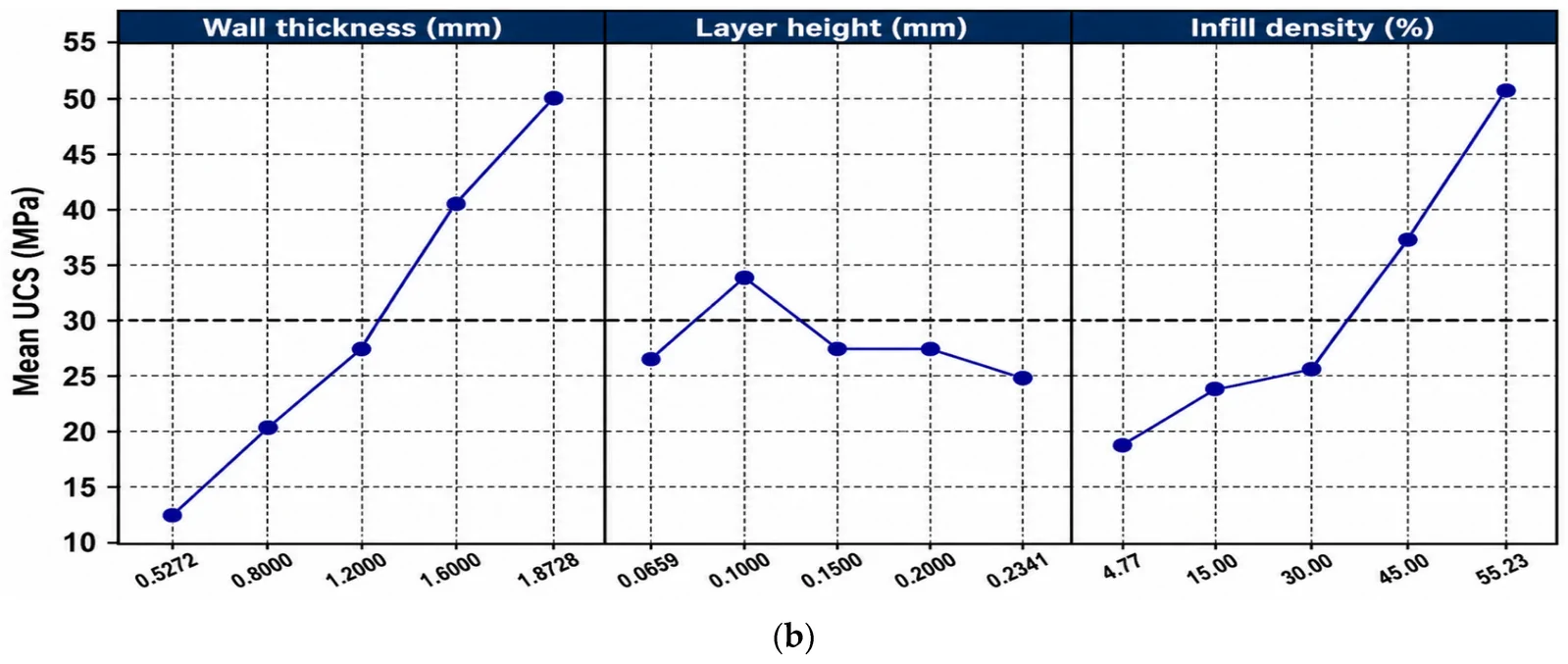
Main effect plots for the UCS of FDM-printed (**a**) ABS and (**b**) ABS-GF samples.

The main effect plots for the UCS of neat ABS shown in Figure 7a show that all three printing parameters considerably influence UCS but with different sensitivities and trend profiles. Wall thickness (*W_t_*) exhibits non-linear behaviour, as it increases UCS gradually from ~15 MPa to a peak of ~30 MPa for the range of 0.5272–1.60 mm before collapsing sharply to ~12.8 MPa at 1.87 mm. This behaviour indicates the existence of an optimum *W_t_*, beyond which residual thermal stresses and inconsistencies between deposited layers may develop, compromising the specimen under compression loading [55]. Specifically, thicker walls require additional deposition time, allowing greater cooling and contraction of previously deposited material, thereby intensifying residual thermal stress and micro-warping at the shell–infill interface [55]. Layer height (*L_h_*) exhibits a fluctuating trend, with UCS values ranging between ~22.5 MPa and ~28.5 MPa across the tested range, confirming its minor influence on ABS under compression. This behaviour arises because the ABS polymer matrix bears the majority of the compression load and is insensitive to variations in interlayer bonding quality [19,30,35]. Contrastingly, the infill density (*I_d_*) sees a steep monotonous rise in the UCS values from ~18.5 MPa to ~37.5 MPa. This represents the largest variation in UCS among all three parameters, confirming *I_d_* as the most influential factor. This increase in UCS is attributed to the absence of internal voids and associated increase in the load-bearing cross-section area with increasing infill fraction [12,13,17]. The main effect plots for the UCS of ABS-GF printed samples shown in Figure 7b represent a different and more pronounced sensitivity profile when compared to its counterpart. Wall thickness (*W_t_*) exhibits a strong, consistent, and nearly linear increase in UCS across the entire tested range, rising from ~15 MPa to ~54 MPa. This trend is far steeper than the one observed for ABS. This behaviour is due to the accumulation of glass-fibre-laden perimeter tracks, where increasing *W_t_* results in deposition of more aligned fibres [17,18]. Layer height (*L_h_*) in ABS-GF exhibits a broadly oscillatory trend similar to that observed in ABS, although with greater variation in UCS. The UCS reaches a maximum of ~35 MPa, and thereafter gradually decreases to ~26.5 MPa, resulting in a modest overall range, classifying *L_h_* as the least-dominant parameter. The higher sensitivity of *L_h_* in ABS-GF reflects that fibre continuity and orientation across interlayer boundaries are more susceptible to changes in deposition conditions [16,17,18]. It is important to note that variations in *L_h_* tend to modify the fibre distribution and influence interlayer fusion, thereby reducing the stress transfer efficiency [18]. Infill density (*I_d_*) produces the most dramatic effect of all parameters in ABS-GF, wherein UCS remains comparatively low and rises from 21 MPa to 39 MPa before exhibiting a near vertical surge to 51 MPa, which accounts to an increase of approximately 59%. This remarkable increase is mainly due to the dense infill, which not only maximizes the material volume within the printed structure but also promotes the formation of a well-connected internal fibre network [10,19]. This material continuity reduces internal voids and improves load transfer between adjacent rasters and fibre reinforcements [17,18]. Together with glass fibre reinforcement, this structural arrangement improves load transfer efficiency, resulting in approximately 2.4 times higher compression strength when compared to ABS, thereby confirming *I_d_* as the most critical parameter for ABS-GF.

### 3.3. Regression Model Analysis for UCS of ABS and ABS-GF

The CCD model was used to correlate the independent parameters (*W_t_*, *L_h_*, *I_d_*) and dependent parameters (UCS) based on experimentation. The regression equations and their summaries developed from experimental input–output data without any bias for UCS with ABS material have been presented in Table 6. The statistical adequacy of the model was evaluated using regression coefficients. The purely linear model (*R*^2^ = 42.90%, and Adj. *R*^2^ = 32.20%) only captures the first-order independent contributions of *W_t_*, *L_h_*, and *I_d_* to UCS. The regression model shows that the relationship between the process parameters and UCS is complex and non-linear and therefore cannot be accurately described by simple linear terms alone. The linear + interaction model (*R*^2^ = 82.89%, and Adj. *R*^2^ = 74.99%) shows significant improvement through the implementation of two-way interaction terms (*A* × *B*, *A* × *C*, *B* × *C*), confirming that parameters do not influence UCS independently but rather influence it through coupled interactions. Notably, the high negative coefficient (−255.4) associated with the interaction terms (*A* × *B*) shows that increasing both *W_t_* and *L_h_* simultaneously has a negative effect on UCS, rather than a positive one. The quadratic model including linear, square, and interaction terms resulted in a regression coefficient *R*^2^ of 0.9538, which clearly indicates the model of best fit. The inclusion of squared terms (*A*^2^, *B*^2^, *C*^2^) captures the non-monotonic and curvilinear behaviour of UCS particularly observed in cases of first increase and subsequent decrease, making the model most reliable for predictive optimization of ABS across the complete experimental range.

In comparison to ABS, the regression models for the UCS of ABS-GF shown in Table 7 reveal a considerably different predictive behaviour, especially with the linear model achieving *R*^2^ = 78.22% and Adj. *R*^2^ = 74.14%. This indicates that the first-order parameter effects are far more dominant in the ABS-GF system. This implies that the ABS-GF model effectively suppresses the complex and non-linear relation of process parameters with UCS, yielding a physically consistent response with improved predictability across the investigated design space. The linear + interaction model for ABS-GF saw a noteworthy improvement in the predictive capability, with an *R*^2^ = 82.89% and Adj. *R*^2^ = 74.99%. Among the interaction terms, the term (*B* × *C*) had a positive coefficient (+3.27), revealing synergetic relation between *L_h_* and *I_d_*. This suggests that high infill density levels and moderate layer heights promote better packing of glass fibres and improve the internal contact area between the deposited layers [18,19,53]. The full quadratic model for the UCS of ABS-GF achieved the highest *R*^2^ = 97.12% and Adj. *R*^2^ = 94.53%, making it the most accurate model amongst both material systems. The model shows a notably large coefficient for the square term *B*^2^, indicating a pronounced influence of layer height on the UCS of ABS-GF. This behaviour is attributed to the greater sensitivity of fibre orientation, continuity, and interlayer bonding between deposited layers, effects that are largely absent in the unfilled ABS.

### 3.4. ANOVA for UCS of ABS and ABS-GF

The Analysis of Variance (ANOVA) results for ABS have been presented in Table 8 and extend the regression model findings [38]. The regression model was found to be highly significant with values of F = 22.95 and *p* = 0.000. This means that all of the selected process parameters collectively account for a statistically significant proportion of the variance in UCS. *W_t_* emerged as the linear parameter with the most statistical significance (F = 53.72, *p* = 0.000), demonstrating that the addition of perimeter tracks (extruded filament paths) directly improves the structural continuity and resistance to compression loading [32]. *L_h_* was the second most statistically significant linear term (F = 28.17, *p* = 0.000), confirming the regression finding that increasing *L_h_* adversely effects UCS due to the formation of coarser layers and diminished inter-layer contact between successively deposited layers [35,36]. The statistical significance of linear *I_d_* was found to be the least (F = 6.19, *p* = 0.032) when compared to the other linear terms, which appears to be counterintuitive provided its dominant role in the main effect plots. However, this contribution of *I_d_* becomes increasingly influential through the quadratic term (*I_d_* × *I_d_*), which exhibited values of F = 15.33 and *p* = 0.003. This indicates that the behaviour of *I_d_* on the UCS of neat ABS is inherently curvilinear rather than purely linear, signifying that increasing infill beyond a specific threshold would result in a closure of voids, resulting in a gradual reduction in compressive strength. Amongst the interaction terms, *W_t_* × *L_h_* was most significant with the highest values (F = 58.74, *p* = 0.000), signifying that *W_t_* and *L_h_* exert a coupled influence on the UCS of ABS. Thick walls with coarser layers govern the fusion quality between perimeter tracks, making their interaction highly critical in controlling structural continuity, a key indicator for improving UCS. The interaction term *I_d_* × *L_h_* also showed strong significance (F = 26.69, *p* = 0.000), indicating that finer layers with high infill produced a denser internal lattice that resists compressive loads. The lack-of-fit significance (F = 5.31, *p* = 0.045) proves that the full quadratic model captured dominant trends; however, some non-linear or localized behaviour remains unaccounted for, like the first peak followed by a sudden collapse variation for *W_t_* observed in the main effect plots that a standard quadratic surface cannot fully represent.

The ANOVA results for the UCS of the ABS-GF system presented in Table 9 show that the regression model was again highly significant (F = 37.51, *p* = 0.000). The F-value is comparatively higher than that of the ABS system, confirming that the influence of process parameters is more consistent and predictable in the fibre-reinforced system. However, unlike pure/neat ABS, the linear terms *W_t_* and *L_h_* were statistically insignificant with *p*-values of 0.108 and 0.914, respectively. Their contribution to the UCS of ABS-GF is manifested through their square and interaction terms, i.e., (*W_t_* × *W_t_*) and (*W_t_* × *L_h_*), with *p*-values of 0.004 and 0.011, respectively. This indicates that the *W_t_* influences UCS nonlinearly, whereby progressive accumulation of fibre-laden perimeter tracks enhances the density of the fibre network within the walls [32]. As a critical level of structural percolation is reached, the material exhibits disproportionately higher load-carrying capacity, consequently leading to higher UCS values. The square term associated with infill density (*I_d_* × *I_d_*) exhibited the highest statistical significance (F = 24.64, *p* = 0.001) for the UCS of ABS-GF, consistent with the non-linear trend observed in the main effect plots. This indicates the non-linear influence of infill density due to the percolation of the glass-fibre network within the internal lattice. There is a specific threshold beyond which fibres tend to form a continuous load-bearing structure across the cross-section, resulting in the sudden amplification of compressive load transfer that cannot be captured by a simple linear term [17,18]. Furthermore, the interaction terms (*W_t_* × *I_d_*) and (*I_d_* × *L_h_*) were both significant, confirming that the effect of infill density depends on both *W_t_* and *L_h_*. From a mechanistic perspective, a thicker wall with higher infill density promotes fibre continuity between the perimeter and internal structure, while fine layers with higher infill preserve fibre alignment and promote interlayer bonding, thereby improving load transfer under compression [31,32,33,34]. The lack-of-fit significance (F = 8.02, *p* = 0.02) suggests non-linear behaviour beyond the predictive capability of the quadratic model. This indicates that the UCS variation is more abrupt rather than smooth, which the polynomial regression models can only approximate rather than accurately capture.

**Figure 8:**
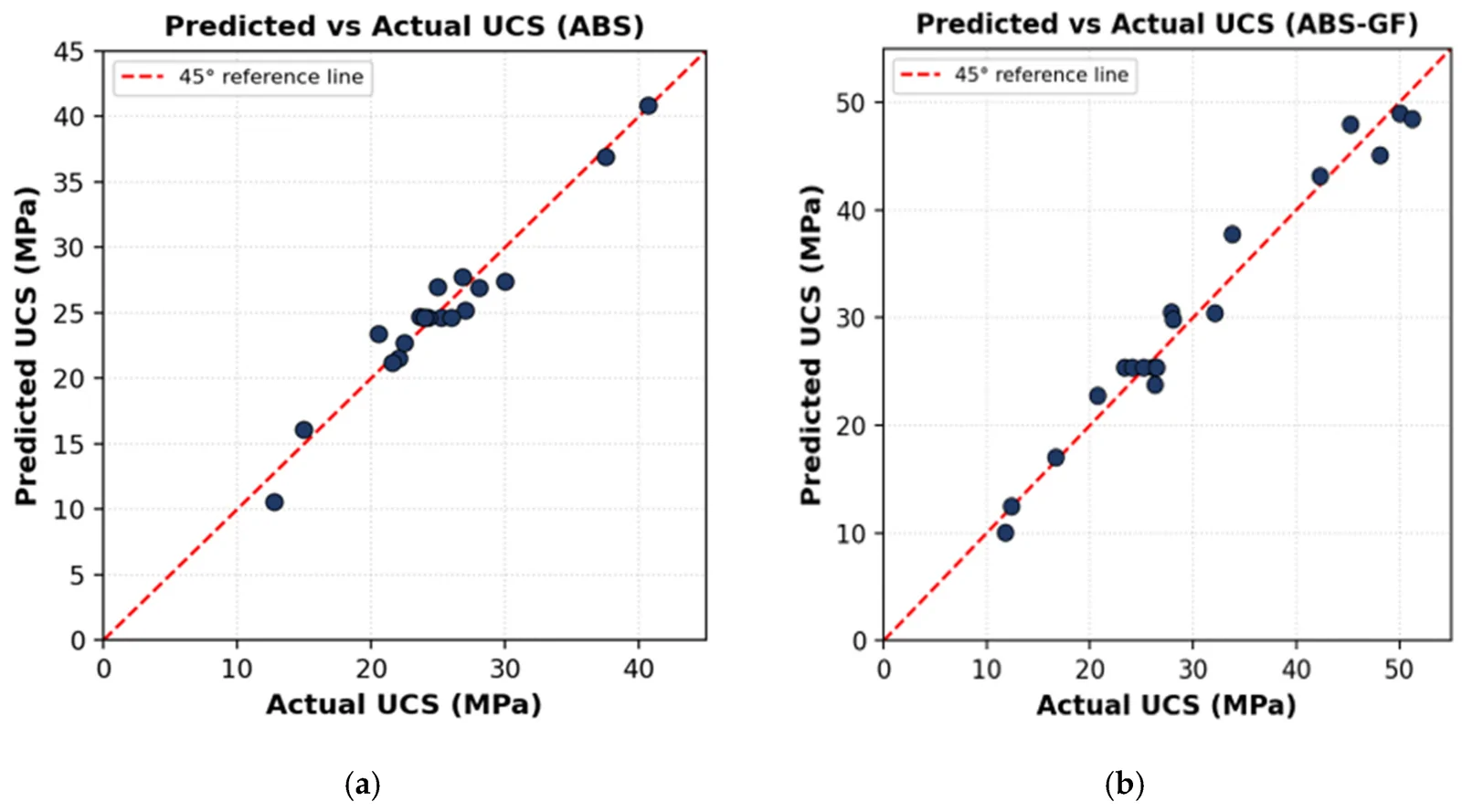
Predicted vs. actual UCS for (**a**) ABS and (**b**) ABS-GF printed parts.

When the insignificant terms were excluded (*p*-value > 0.05) from the regression equations, there was a noticeable decline in the prediction accuracy. Both models were therefore tested for prediction accuracy with the experimental results by including all terms. The line of best fit presented in Figure 8a,b shows all data closely distributed around the reference (y = x) line, indicating excellent agreement between experimental and predicted UCS values. This demonstrates that the developed regression equations are reliable and accurate and could be further used to perform optimization.

### 3.5. Response Surface Analysis for UCS of ABS and ABS-GF

**Figure 9:**
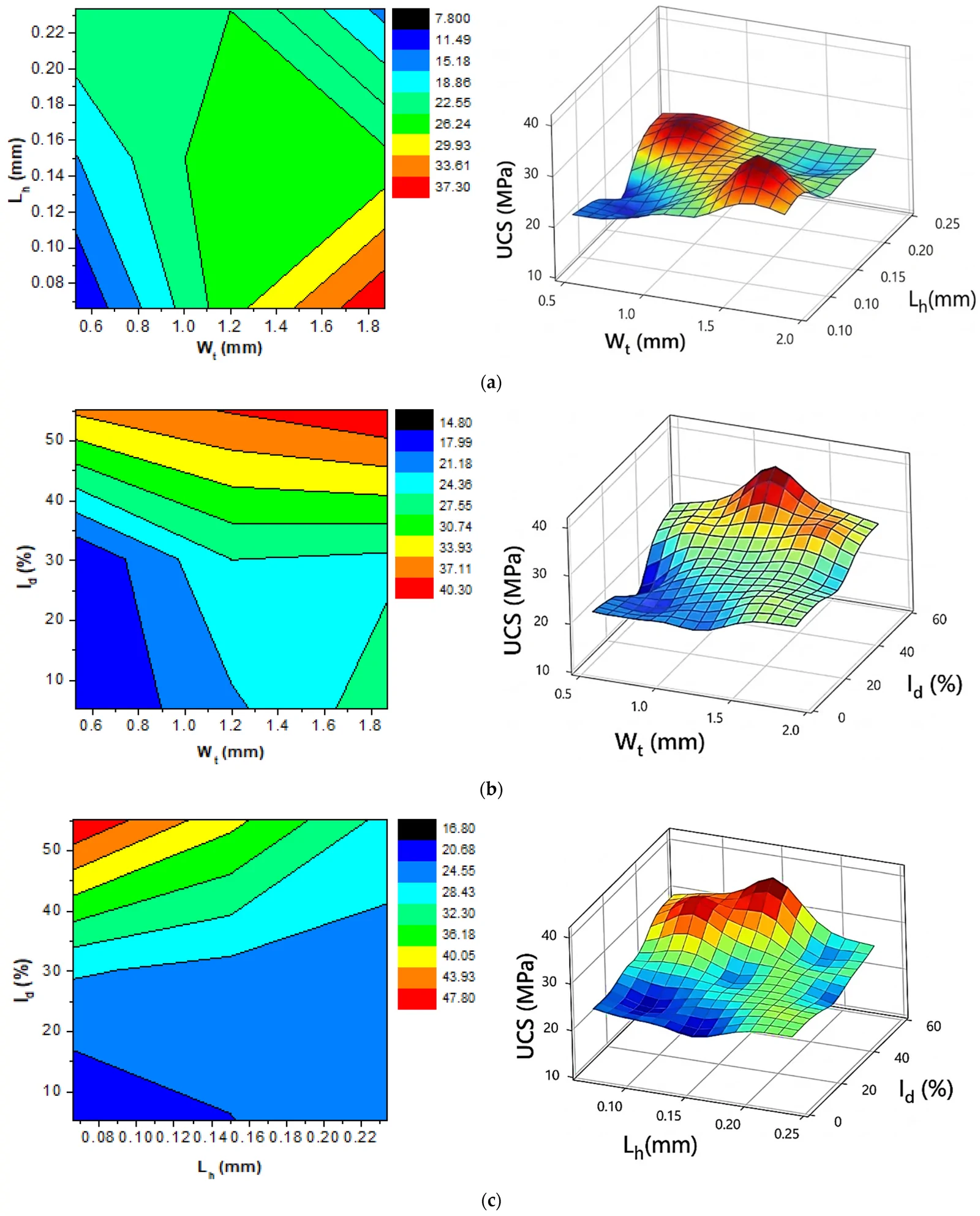
Contour and surface analysis plots for the UCS of FDM-printed ABS samples for interactions (**a**) *W_t_*–*L_h_*, (**b**) *W_t_*–*I_d_*, (**c**) *I_d_*–*L_h_*.

**Figure 10:**
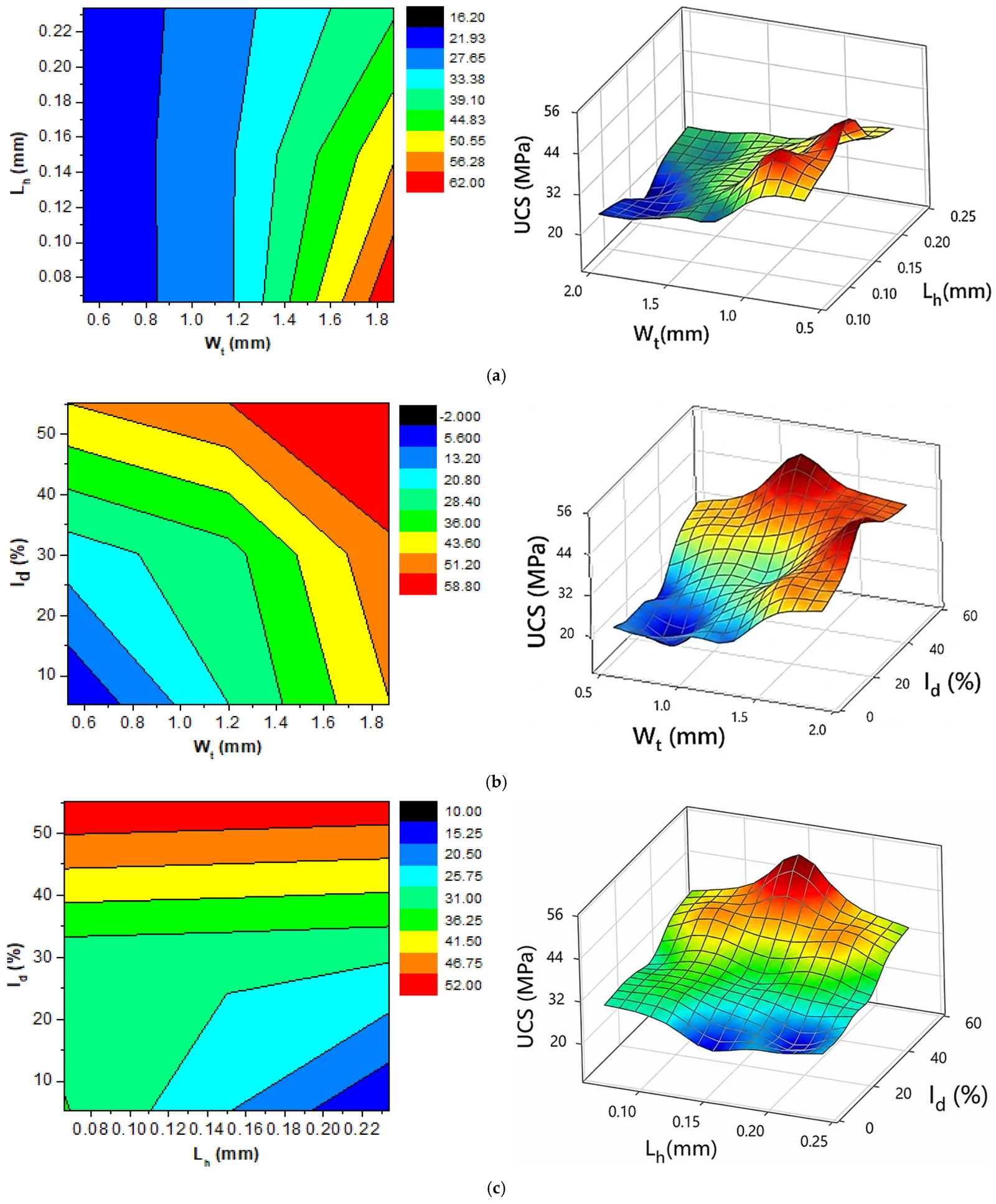
Contour and surface analysis plots for the UCS of FDM−printed ABS−GF samples for interactions (**a**) *W_t_*–*L_h_*, (**b**) *W_t_*–*I_d_*, (**c**) *I_d_*–*L_h_*.

**Figure 11:**
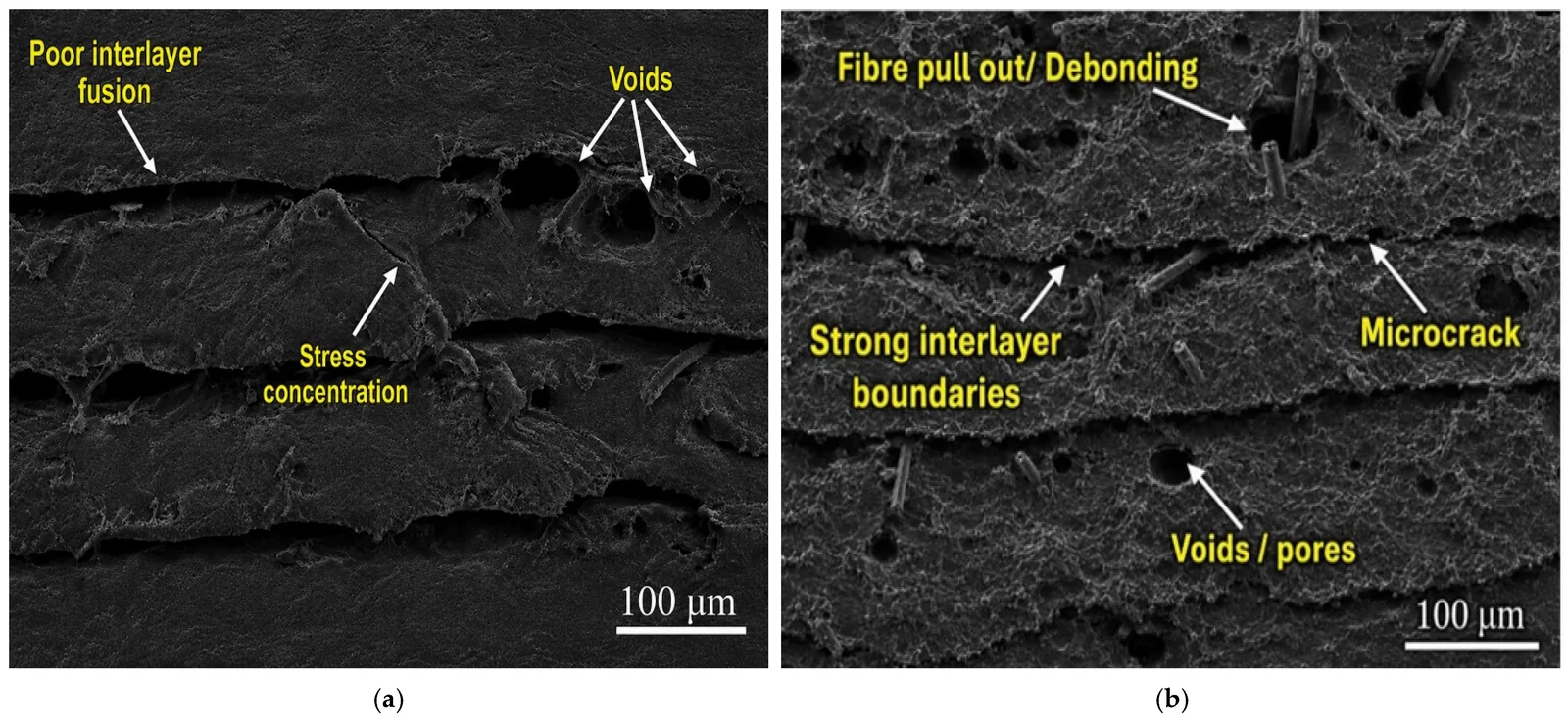
SEM features of neat (**a**) ABS and (**b**) ABS-GF specimen under compression loading.

The contour plots and response surface analysis for the UCS of FDM-printed ABS and ABS-GF specimens with respect to the interaction parameters has been analyzed in Figure 9 and Figure 10, respectively. In Figure 9a, the combined influence of wall thickness (*W_t_*) and layer height (*L_h_*) on the UCS of FDM-printed ABS samples is analyzed. Both parameters, i.e., *W_t_* and *L_h_*, exhibit a complex, non-linear interaction with UCS. At low values of *W_t_* (≈0.08–0.12 mm), the compression strength remained mostly modest for most *L_h_* values, indicating that extremely thin walls often fail to provide structural integrity under compression loading. This is primarily due to material discontinuity and weak bonding between ABS layers during FDM printing [22]. The inadequate fusion between successive ABS layers during printing tends to create voids and discontinuities within the structure, thereby developing stress concentration sites which reduce the specimen’s ability to uniformly transfer load [22,35]. At moderate *W_t_* values (≈0.14–0.16 mm), the UCS reaches a peak for *L_h_* ranging between 1.0–1.2 mm, indicating optimal printing conditions where layer adhesion and material deposition are effectively balanced. Following this, any further increase in the values of *L_h_* beyond 1.2 mm cause a steady decline in the UCS regardless of *W_t_*, which could be attributed to weaker interlayer fusion and greater void formation between deposited layers [32]. Therefore, the interaction between *W_t_* and *L_h_* indicates that UCS is not governed by either parameter individually; instead, their coordinated selection determines the best mechanical performance of the printed part. Figure 9b presents the combined influence of wall thickness (*W_t_*) and infill density (*I_d_*) on the UCS of FDM-printed ABS samples. Here, *I_d_* emerges as the most dominant parameter, as it significantly increases the UCS by about 55% for all levels of *W_t_*, reflecting the importance of internal material volume fraction in resisting compressive loads. Low values of *I_d_* develop sparse internal lattices producing structurally hollow specimens that provide minimal resistance to compressive loading and hence are prone to early buckling or the collapse of internal struts, irrespective of the wall thickness [13,32,36]. *W_t_*, on the other hand, plays a considerate role in amplifying UCS at any given value of *I_d_*, as thicker walls improve the load-bearing capacity and enhance the structural continuity between the outer boundary and internal infill network [13]. The synergetic interaction is highest at higher values of *W_t_* and *I_d_*, wherein dense internal structures and thicker walls collectively create a solid cross-section that maximizes compression strength. Figure 9c shows the combined interaction of layer height (*L_h_*) and infill density (*I_d_*) on the UCS of FDM-printed ABS. Across the entire range of *L_h_*, an increasing *I_d_* significantly increases the UCS due to stronger and denser internal lattices that improve resistance to internal buckling under axial compression [36]. However, at higher values of *I_d_*, *L_h_* exerts a notable influence, with lower layer heights yielding maximum UCS values of ~44–48 MPa. Finer layers promote stronger interlayer bonding through thermal re-melting across the contact area between successive deposited layers, resulting in a more homogeneous and void-free microstructure [13,15,18]. Beyond *L_h_* ~ 0.15 mm, the UCS declines progressively for higher values of *I_d_*, which could be attributed to thicker layers generating interlayer voids and weaker fusion zones, thereby increasing the anisotropy in the printed structure that weakens the specimen under compression loading [36]. At low *I_d_*, the effect of *L_h_* is minimal, as the internal structure is already sparse with significant structural weakness. Consequently, irrespective of the interlayer bonding, the specimen is unable to effectively sustain the applied load. This interaction, therefore, creates an optimal processing window wherein maximum UCS is achieved at high *I_d_* and low *L_h_*. The SEM features of the ABS specimen under compression loading are shown in Figure 11a.

The contour and surface plots showing the interaction of wall thickness (*W_t_*) and layer height (*L_h_*) on the UCS of FDM-printed glass-fibre-reinforced ABS (ABS-GF) is shown in Figure 10a. It is observed that when the *W_t_* was varied from ≈0.08–0.22 mm, the UCS sharply increased to a peak value of ~60 MPa for all values of *L_h_*, defying its critical role in load-bearing FDM components. This strong dependency of *W_t_* on ABS-GF was not seen in the FDM-printed neat ABS samples and could be mainly because of the glass fibre reinforcement. With increasing *W_t_*, a higher number of glass fibres is deposited on the ABS plastic and contribute to the effective load-bearing capacity and improved resistance to compressive failure [17,18]. *L_h_*, on the other hand, becomes apparent at higher levels of *W_t_*. When the values of *L_h_* are low (~0.10–0.14 mm), the UCS is slightly superior due to finer deposition and uniform fibre distribution across interface layers, significantly reducing voids and thereby preserving the microstructure of ABS-GF [10,17]. However, for *L_h_* values in the range of (~0.18–0.20 mm), the interlayer bonding weakens due to thermal re-melting, causing a considerable drop in the UCS, even at higher *W_t_* [33,35,36]. When compared to neat ABS, the ABS-GF printed parts exhibited higher UCS, with *W_t_* being the dominant parameter. Therefore, optimization of *W_t_* is crucial when processing short fibre-filled composite filaments via FDM. The combined influence of wall thickness (*W_t_*) and infill density (*I_d_*) on the UCS of FDM-printed ABS-GF can be seen in Figure 10b. Infill density once again emerged as the most influential parameter, substantially increasing UCS across all levels of *W_t_*, mainly driven by progressive increase in the internal volume that directly supports load-bearing capacity and avoids void formation [32,36]. On the other hand, *W_t_* becomes increasingly influential, as it significantly improved the UCS when increased from 0.05 to 0.18 mm. This trend is particularly pronounced in ABS-GF as compared to ABS, as each thickness perimeter deposits glass-fibre-reinforced material, which contributes to the compression loading resistance. The synergetic interaction of *W_t_* and *I_d_* results in the highest UCS when both parameters are set at high levels. A higher infill density creates a dense internal structure, while the fibre-reinforced perimeter walls create a structurally solid cross-section that yields a peak UCS of ~56–59 MPa. Conversely, low *W_t_* and *I_d_* make the specimen structurally deficient, resulting in low UCS values. When compared to the ABS specimen, the interaction between *W_t_* and *I_d_* is less symmetric in ABS-GF samples. The combined interaction of layer height (*L_h_*) and infill density (*I_d_*) is shown in Figure 10c. Just like the previous cases, an increase *I_d_* saw a steep rise in the UCS values (~52–55 MPa) for all values of *L_h_*, mainly due to denser infill structures. However, varying *L_h_* alone for any fixed value of *I_d_* (below ~40%) produces a negligible change in UCS, meaning interlayer bonding is the limiting factor at low infill densities irrespective of whether the layers are finely or coarsely deposited. For values of *I_d_* ranging between 40 and 60%, minor variations in the UCS are observed for changes in *L_h_*. Finer layer heights (~0.08–0.15 mm) yield measurable UCS values when compared to coarser layer heights (~0.15–0.23 mm). This is because higher infill densities along with thinner layer heights develop stronger interlayer bonds with uniform distribution of glass fibres across layer interfaces, reducing void formation and thereby promoting the overall compressive integrity of the specimen. Comparatively, in ABS-GF, the *L_h_* is more sensitive at higher values than *I_d_*. This is because increasing the layer height affects the alignment and distribution of glass fibres along the deposition direction, reducing the effectiveness of the fibre network in resisting compressive loads. The SEM features of the ABS specimen under compression loading are shown in Figure 11b.

### 3.6. Summary of the Optimization Algorithms

The set of optimal FDM printing parameters that maximize the UCS of both the ABS and ABS-GF systems was predicted using three algorithms (FA, BB-BS, and GWO). The quadratic equations for ABS and ABS-GF presented in Table 6 and Table 7, respectively, were used as the objective function for the optimization of maximum compression strength. The search algorithms used the design space with upper and lower bounds of the printing parameters, defined in Table 4. The performance of all three algorithms was assessed using computational time and solution accuracy. To ensure consistent termination criterion, common algorithm parameters such as population size and iterations were kept constant at 100 and 1000, respectively. Each algorithm was independently tested 10 times for stochastic variability. The best-performing solutions for the UCS of ABS and ABS-GF systems corresponding to fitness value and computation time were recorded and are presented in Table 10 and Table 11, respectively. It is important to appropriately tune the algorithm’s specific parameters, such as the convergence constant for GWO, exploration factor for BB-BC, and the randomness, absorption coefficient, and randomness reduction for FA, as they strongly influence the efficiency and effectiveness of the optimization process [45,46,47,48,49,50]. The tuning for each algorithm-specific parameter was primarily conducted to maximize UCS, and the optimal process parameters for subsequent trials were selected based on the results obtained from each algorithm run. The optimal process parameter conditions were therefore evaluated after fixing the algorithm-specific parameters (population size = 100 and iteration = 1000) to obtain maximum UCS. The optimal conditions and fitness values for all three algorithms, i.e., FA, BB-BC, and GWO, for the UCS of both ABS and ABS-GF systems are presented in Table 10 and Table 11, respectively. It is important to note that for both cases, the GWO and FA resulted in almost identical optimal conditions, as compared to that obtained for BB-BC. The GWO algorithm yielded the best fitness values of 97.15% and 98.48% for the ABS and ABS-GF systems, respectively. The GWO heavily relies on a constant convergence parameter, which controls the search radius of the grey wolves, enabling effective trade-off between exploration and exploitation and leading to consistent and accurate predictions [49,50]. Confirmation experiments were conducted to validate the optimal printing parameter conditions. The ABS system yielded an experimental UCS value of 44.5 MPa at optimal conditions of *W_t_* = 1.813 mm, *L_h_* = 0.089 mm, and *I_d_* = 43.219. Similarly, for the ABS-GF system, the experimental UCS value was 57.2 MPa for optimal conditions of *W_t_* = 1.784 mm, *L_h_* = 0.068 mm, and *I_d_* = 41.483. A PC equipped with MATLAB R2025a (Version 25.1) software was used to compute the UCS of the ABS and ABS-GF systems. The system configuration included a 4 GB RAM and an Intel Core i3 processor @ 1.2 GHz CPU. The computational time required to obtain the maximum fitness values was found to be equal to ~11 s, ~1.5 s, and ~2 s for FA, BB-BC, and GWO, respectively. In both cases, the BB-BC algorithm exhibited the lowest computational time compared to GWO and FA, which may be attributed to simpler parameter tuning involving a single algorithm-specific parameter that improves exploration and search mechanisms [46,47]. However, the simplicity of BB-BS limits its search capabilities, making it susceptible to getting trapped at local minima in a complex multi-dimensional space [47]. The FA, on the other hand, employs a light intensity-based attraction mechanism, which adversely influences the search in high-dimensional spaces due to the interaction between all fireflies, causing additional computational burden [45]. Among all three algorithms, GWO is recommended for obtaining maximum UCS due to effective balance between exploration and exploitation, achieved through the social hierarchy and hunting behaviour of grey wolves. This exploration–exploitation balance prevents the algorithm from stagnating in local optima within non-linear, multimodal design spaces [56,57,58]. This eventually enables the algorithm to achieve superior solution quality with reasonable computational time.

## 4. Conclusions

The present study investigates the influence of three FDM printing parameters, namely, wall thickness, layer height, and infill density, on the compressive behaviour of ABS and fibre-reinforced ABS printed parts. The RSM-based CCD with 20 experimental runs was employed to develop predictive regression models to quantify the relationship between process parameters and ultimate compressive strength, while metaheuristic algorithms, namely, FA, BB-BC and GWO, were employed to determine the optimal printing conditions. Based on the experimental and numerical analyses, the following conclusions are drawn:

- The reinforcement of ABS with glass fibres significantly improved the compression strength of the FDM-printed parts, demonstrating superior load-carrying capacity and deformation characteristics when compared to neat ABS.
- From the main effect plots, infill density was found to be the most influential parameter, as it reduced internal porosity, increased the load-bearing cross-sectional area, and improved stress transfer through a consistent internal structure. In ABS-GF, higher infill density further improved the fibre network continuity, resulting in superior compressive performance.
- The RSM-based quadratic equations were able to accurately predict compression strength for both materials, achieving coefficients of determination of approximately 95.4% and 97.1% for the ABS and ABS-GF, respectively. This demonstrates the ability of RSM to capture non-linear relationships between FDM process parameters and mechanical performance.
- The optimization algorithms (FA, BB-BC, and GWO) successfully identified the optimal combinations of process parameters for maximizing compressive strength. Amongst the algorithms, GWO provided the best optimization performance exhibiting stable convergence within less computational time.
- The integrated framework combining experimental testing, RSM, and metaheuristic algorithms provides an efficient and reliable methodology for optimizing FDM printing parameters, thereby reducing experimental effort and improving manufacturing efficiency. Additionally, this framework could be extensively applied to other material systems, including fibre-reinforced polymers such as PLA-GF, ABS-CF, PLA-CF, etc., provided the process parameters, material systems, and response variables are appropriately predefined.

## Figures and Tables

**Table 1:** Summary of recent RSM and neural network applications in FDM-printed parts.

Study	Material	Process Parameters	Technique	Predicted Output	Predicted Performance
Singh et al. [28]	PLA	Nozzle diameter, build orientation, raster pattern, layer height, print speed	Feed-forward back-propagation NN	UTS and *Ra*	*R*^2^ = 0.99366
Malleswari et al. [29]	PLA	Bed temperature, print speed, layer thickness, orientation angle	RSM and ANN	*Ra*	*R*^2^ = 0.99353 (RSM); *R*^2^ = 0.99272 (ANN)
Hamed [30]	ABS	Layer thickness, printing speed, direction angle, nozzle temperature	ANN with regression	UTS	MAPE = 5.518% (ANN); MAPE = 19.37% (Regression)
Zathry et al. [31]	PLA	Extrusion temperature, layer height, printing speed	ANN (3-10-1) architecture	*Ra*	*R*^2^ > 0.99; MAPE = 1.23%
Karadag and Ulkir [32]	PLA, PETG, TPU	Material type, layer thickness, infill density, infill pattern, wall thickness	Feed-forward ANN & RSM	Dimensional accuracy and *Ra*	*R*^2^ > 0.96 across geometries
Contuzzi et al. [33]	PLA/wood bio-composite	Scanning speed, printing temperature, layer height	RSM and ANN	Dimensional accuracy and *Ra*	*R*^2^ > 0.99, ANN reported as better than regression
Zurnacı & Alıcıoğlu et al. [34]	PLA	Raster angle, infill type, infill rate	Taguchi L18 orthogonal array	UTS and impact strength	MAPE = 3.89% (UTS); MAPE = 9.57% (Impact strength)
Venkatraman & Raghuraman et al. [35]	ABS	Infill percentage, layer height, bed temperature	RSM-based BBD	Micro-hardness and *Ra*	*R*^2^ > 0.99 for both micro-hardness and Ra
Gao et al. [36]	PLA	Infill pattern, printing speed, printing temperature, layer thickness	RSM-based CCD and Taguchi L27 orthogonal array	UTS and UCS	Prediction deviations = 4.22% (UTS); 1.89% (UCS)

**Table 2:** Mechanical properties of glass fibre and ABS.

Properties	Units	E-Glass Fibre	ABS
Tensile strength	MPa	3100–3800	≥43
Young’s modulus	GPa	80–83	1.79–3.2
Impact strength (IZOD, 23 °C)	J/m	-	≥108
Flexural strength and modulus	MPa	-	≥70 & ≥2300
Elongation at break	(%)	4.8	≥30

**Table 3:** Physical properties of glass fibre and ABS.

Properties	Units	E-Glass Fibre	ABS
Melting point	°C	1150–1250	~200
Density	g/cm^3^	2.62	1.04–1.07
Grams per square metre	-	300	-

**Table 4:** Experimental variables and levels.

Parameters	Symbol	Units	−1.682 (Axial Low)	(−1) Factorial Low	(0) Centre	(+1) Factorial High	+1.682 (Axial High)
Wall thickness (*W_t_*)	A	(mm)	0.5272	0.8	1.2	1.6	1.8728
Layer height (*L_h_*)	B	(mm)	0.0659	0.1	0.15	0.20	0.2341
Infill density (*I_d_*)	C	(%)	4.77	15	30	45	55.23

**Table 5:** Experimental results as per CCD matrix.

Run	Uncoded			Coded			Ultimate Compression Strength (MPa)	
	A (mm)	B (mm)	C (%)	*W_t_* (mm)	*L_h_* (mm)	*I_d_* (%)	ABS	ABS-GF
1	−1	−1	−1	0.8	0.1	15	12.72	12.42
2	1	−1	−1	1.6	0.1	15	29.97	48.08
3	−1	1	−1	0.8	0.2	15	27.02	11.76
4	1	1	−1	1.6	0.2	15	22.02	27.87
5	−1	−1	1	0.8	0.1	45	28.08	32.10
6	1	−1	1	1.6	0.1	45	40.66	45.23
7	−1	1	1	0.8	0.2	45	26.79	33.79
8	1	1	1	1.6	0.2	45	21.56	42.31
9	−1.682	0	0	0.5272	0.15	30	14.92	16.68
10	1.682	0	0	1.8728	0.15	30	23.63	50.02
11	0	−1.682	0	1.2	0.0659	30	24.95	28.02
12	0	1.682	0	1.2	0.2341	30	22.50	26.29
13	0	0	−1.682	1.2	0.15	4.77	20.55	20.76
14	0	0	1.682	1.2	0.15	55.23	37.47	51.20
15	0	0	0	1.2	0.15	30	25.23	26.13
16	0	0	0	1.2	0.15	30	24.00	23.29
17	0	0	0	1.2	0.15	30	24.23	26.29
18	0	0	0	1.2	0.15	30	24.00	24.10
19	0	0	0	1.2	0.15	30	24.00	25.18
20	0	0	0	1.2	0.15	30	26.01	26.45

**Table 6:** Regression equations for ABS.

Linear	UCS = 13.14 + (6.41 × *A*) − (25.4 × *B*) + (0.2664 × *C*)	*R*^2^ = 42.90%, and Adj. *R*^2^ = 32.20%
Linear + Interaction	UCS = 57.8 + (48.3 × *A*) + (418.8 × *B*) + (1.098 × *C*) − (255.4 × *A* × *B*) − (0.119 × *A* × *C*) − (4.59 × *B* × *C*)	*R*^2^ = 82.89%, and Adj. *R*^2^ = 74.99%
Linear + Square + Interaction	UCS = −63.2 + (70.76 × *A*) + (409.9 × *B*) + (0.580 × *C*) − (9.36 × *A*^2^) + (30 × *B*^2^) + 0.00864 × *C*^2^) − (255.4 × *A* × *B*) − (0.119 × *A* × *C*) − (4.592 × *B* × *C*)	*R*^2^ = 95.38%, and Adj. *R*^2^ = 91.22%

**Table 7:** Regression equations for ABS-GF.

Linear	UCS = −8.35 + (23.70 × *A*) − (36.6 × *B*) + (0.510 × *C*)	*R*^2^ = 78.22%, and Adj. *R*^2^ = 74.14%
Linear + Interaction	UCS = −43.4 + (65.2 × *A*) + (46 × *B*) + (0.772 × *C*) − (151.0 × *A* × *B*) − (0.627 × *A* × *C*) + (3.27 × *B* × *C*)	*R*^2^ = 87.07%, and Adj. *R*^2^ = 81.10%
Linear + Square + Interaction	UCS = −5.0 + (25.0 × *A*) − (12 × *B*) − (0.191 × *C*) + (16.76 × *A*^2^) + (196 × *B*^2^) + (0.01605 × *C*^2^) − (151.0 × *A* × *B*) − (0.627 × *A* × *C*) + (3.27 × *B* × *C*)	*R*^2^ = 97.12%, and Adj. *R*^2^ = 94.53%

**Table 8:** Analysis of Variance (ANOVA) for the UCS of the ABS system.

Source	DF	Adj SS	Adj MS	F-Value	*p*-Value
Regression Model	9	733.963	81.551	22.95	0.000
*W_t_*	1	190.929	190.929	53.72	0.000
*L_h_*	1	100.113	100.113	28.17	0.000
*I_d_*	1	22.015	22.015	6.19	0.032
*W_t_* × *W_t_*	1	32.319	32.319	9.09	0.013
*L_h_* × *L_h_*	1	0.080	0.080	0.02	0.884
*I_d_* × *I_d_*	1	54.488	54.488	15.33	0.003
*W_t_* × *L_h_*	1	208.754	208.754	58.74	0.000
*W_t_* × *I_d_*	1	4.078	4.078	1.15	0.309
*L_h_* × *I_d_*	1	94.875	94.875	26.69	0.000
Error	10	35.541	3.554	–	–
Lack-of-Fit	5	29.908	5.982	5.31	0.045
Pure Error	5	5.633	1.127	–	–
Total	19	769.504	–	–	–

**Table 9:** Analysis of Variance (ANOVA) for the UCS of the ABS-GF system.

Source	DF	Adj SS	Adj MS	F-Value	*p*-Value
Regression Model	9	2574.17	286.019	37.51	0.000
*W_t_*	1	23.74	23.743	3.11	0.108
*L_h_*	1	0.09	0.093	0.01	0.914
*I_d_*	1	2.39	2.386	0.31	0.588
*W_t_* × *W_t_*	1	103.61	103.609	13.59	0.004
*L_h_* × *L_h_*	1	3.47	3.467	0.45	0.515
*I_d_* × *I_d_*	1	187.91	187.905	24.64	0.001
*W_t_* × *L_h_*	1	72.95	72.945	9.57	0.011
*W_t_* × *I_d_*	1	113.27	113.274	14.86	0.003
*L_h_* × *I_d_*	1	48.17	48.172	6.32	0.031
Error	10	76.25	7.625	–	–
Lack-of-Fit	5	67.80	13.560	8.02	0.020
Pure Error	9	2574.17	286.019	37.51	0.000
Total	1	23.74	23.743	3.11	0.108

**Table 10:** Optimization of printing parameters for the UCS of ABS using three algorithms.

Algorithms	Specific Parameters	Fitness Value (%)	Computational Time (s)	Optimal Parameter Condition
FA	Absorption coefficient, γ = 1; Randomness, α = 0.9; Randomness reduction, β = 0.6	95.81	11.44	*W_t_* = 1.82 mm; *L_h_* = 0.09 mm; *I_d_* = 43.24
BB-BC	Exploration factor = 15	92.27	1.65	*W_t_* = 1.83 mm; *L_h_* = 0.09 mm; *I_d_* = 44.32
GWO	Convergence constant = 0.5	97.15	2.04	*W_t_* = 1.81 mm; *L_h_* = 0.09 mm; *I_d_* = 43.22

**Table 11:** Optimization of printing parameters for UCS of ABS-GF using three algorithms.

Algorithms	Specific Parameters	Fitness Value (%)	Computational Time (s)	Optimal Parameter Condition
FA	Absorption coefficient, γ = 1; Randomness, α = 0.9; Randomness reduction, β = 0.6	95.91	10.68	*W_t_* = 1.78 mm; *L_h_* = 0.07 mm; *I_d_* = 41.47
BB-BC	Exploration factor = 15	92.48	1.59	*W_t_* = 1.76 mm; *L_h_* = 0.07 mm; *I_d_* = 42.12
GWO	Convergence constant = 0.5	98.48	2.01	*W_t_* = 1.78 mm; *L_h_* = 0.07 mm; *I_d_* = 41.48

## Data Availability

The acquired and presented data is available on reasonable request.

## Abbreviations

The following abbreviations are used in this manuscript:

AM	Additive Manufacturing
FDM	Fused Deposition Modelling
ABS	Acrylonitrile Butadiene Styrene
ABS-GF	Short Glass-Fibre-Reinforced ABS
GF	Glass Fibre
CF	Carbon Fibre
ABS-CF	Carbon Fibre-Reinforced ABS
PLA	Polylactic Acid
PLA-CF	Carbon Fibre-Reinforced PLA
PETG	Polyethylene Terephthalate Glycol
UCS	Ultimate Compressive Strength
RSM	Response Surface Methodology
CCD	Central Composite Design
FA	Firefly Algorithm
BB-BC	Big Bang–Big Crunch algorithm
GWO	Grey Wolf Optimization
STL	Standard Tessellation Language
DF	Degrees of Freedom
SS	Sum of Squares
MS	Mean Square
